# Estimating Alpine Skiers’ Energetics and Turn Radius Using Different Morphological Points

**DOI:** 10.3389/fphys.2018.01541

**Published:** 2018-11-13

**Authors:** Frédéric Meyer, Fabio Borrani

**Affiliations:** Institute of Sport Science, University of Lausanne, Lausanne, Switzerland

**Keywords:** centre of mass, potential energy, kinetic energy, GNSS, giant slalom

## Abstract

Alpine ski analysis has always been very challenging, mainly due to the environmental conditions, large field and rapid and dynamic skiers’ movements. Global navigation satellite system (*GNSS*) offers a solution adapted to outdoor testing, but the relationship between the point where the antenna is attached and the real centre of mass (*CoM*) position is still unknown. This article proposes to compare different points of the body used to quantify the performance of alpine skiers. 3D models of seven elite skiers performing giant slalom (*GS*) were built using multiple camera system and dedicated motion tracking software. *CoM* as well as pelvis, head and feet trajectories were deduced from the data. The potential and kinetic energies corresponding to these points were calculated, as well as the evolution of the turn radius during the turn cycle. Differences between values given by the *CoM* and the other morphological points were analyzed. The pelvis offered the best estimation of the *CoM*: No differences were found for the biomechanical parameters, except for the kinetic energy, where 2% of the turn cycle had significant different values. The head was less accurate compared to the pelvis, showing significant differences with *CoM* between 7 and 20% of the turn cycle depending on the parameter. Finally, the feet offered the worst results, with significant differences between 16 and 41% of the turn cycle. Energies and turn radius calculated by using pelvis in place of *CoM* offered similar patterns, allowing the analysis of mechanical and dissipation energy in *GS*. This may potentially enable easier testing methods to be proposed and tested.

## Introduction

Human movement analyses are usually based on the body centre of mass *(CoM)* position determination. Mechanics of different sports have widely been studied, showing the necessity to calculate the *CoM* with a good accuracy to perform precise analysis [e.g., walking (Cavagna et al., 1963; Saibene and Minetti, 2003; Willems et al., 1995), running (Kyröläinen et al., 2001), cycling (Chèze et al., 1995)]. However, *CoM* calculations usually require large infrastructures such as 3D camera system (Richards, 1999) or a force platform (Barbier et al., 2003). Kinematic arms (Belli et al., 1993) and global navigation satellite system (*GNSS*) (Terrier et al., 2005) have also been used in running and walking analysis, but these methods use a point situated on the back of the subject to approximate the *CoM*. Slawinski et al. (2004) analyzed the use of a lumbar point for the estimation of potential and kinetic mechanical power in running. With this method, they found an overestimation of the kinetic power and underestimation of the potential power. Nevertheless, results obtained by using either a fixed point on the back or the *CoM* were well correlated. Gard et al. (2004) compared three methods (i.e., force platform, marker on the sacrum and full body model) to determine vertical displacement of the *CoM* during walking. They highlighted an overestimation of the vertical displacement of the *CoM* with the sacrum marker. In alpine skiing, the *CoM* has also been used as a reference to perform technical analysis (Kagawa, 2001; Schiefermüller et al., 2005), trajectories and speed analysis (Lešnik and Zvan, 2003) and to analyze energy balance of skiers performing turns both in giant slalom (*GS*) (Supej et al., 2005; Supej, 2008) and in slalom (Reid et al.2009). More recently, Fasel et al. (2016) used both *GNSS* and inertial sensors to determine *CoM* in alpine skiing. An accuracy and precision of 0.08 and 0.04 m respectively were reported for the *CoM* position.

Multiple camera systems are commonly used to reconstruct 3D models of the athlete, and *CoM* is then calculated, with de Leva adjustments (De Leva, 1996), using mathematical models of the body based on Hanavan (1964), Clauser et al. (1969), or Zatsiorsky (1983). However, this method only enables the recording of a small acquisition volume (usually one or two gates) and suffers from the approximation induced by the model. Alternatively, the use of low cost, high accuracy *GNSS* have expanded, allowing analyzing trajectories during a whole run (Waegli and Skaloud, 2007; Gomez-Lopez et al., 2009; Waegli and Skaloud, 2009; Waegli et al., 2009). However, since the *CoM* is not a fixed body point, the link between the antenna trajectory and the real *CoM* of the skier need to be determined. Gilgien et al. (2013) used the pendulum principle to estimate the distance between the real *CoM* position and the position given by a *GNSS* antenna placed on the helmet. Another solution could be to place the antenna to different positions. Therefore, the aim of this work was to compare the use of either the *CoM* or other morphological points to determine delta of potential energy *(*Δ*Epot)*, kinetic energy *(Ekin)* and turn radius *(Trad)* of alpine skiers performing *GS*.

## Materials and Methods

### Participants

Seven European Cup and FIS racers [mean ± standard deviation *(SD):* body mass 98.8 ± 9.1 kg; height 1.82 ± 0.07 m; *GS* FIS points 26.45 ± 14.58] participated in the study. All participants were healthy males without any joint motion problems (World Medical Association, 2013).

### Experimental Design and Setting

**FIGURE 1:**
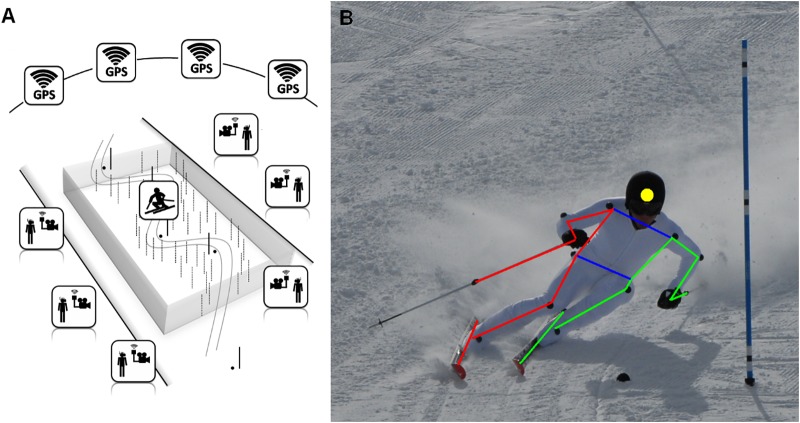
**(A)** Slope setup showing the cameras, the gates and the reference points positions, **(B)** skier suit, markers and body segments.

A *GS* run was set up with a total of six gates, with a linear gate distance of 24 m and a lateral offset of 9 m. The first three gates were used to initiate the rhythm, and the next three were recorded. The slope angle was approximately 22 degrees. Six panning and tilting cameras, 1004^∗^1004 pixels resolution, 48 Hz (PiA1000, Basler, Switzerland) were positioned around the *GS* run, about 35 m from the center of the zone of acquisition (i.e., video captured). Each camera was mounted on a special tripod head, especially built to always keep the sensor center of the camera at the same 3D coordinate, even when the camera was rotated to track the skier. Reference markers mounted on poles were positioned around the run to act as calibration and reference points for the panning and tilting reconstruction. The capture volume was around 60 ^∗^ 20 ^∗^ 2 m (Figure 1A). The positions of each reference marker, gate and camera were measured with a reflectorless total station *(theodolite + laser range finder, LQTS-522D, Longqiang, China*). The cameras’ 3D coordinates were calculated as the median of two points on either side of the tilting axis of the camera. Each camera was connected with Gigabit Ethernet to a dedicated laptop which directly recorded the frames in the RAM memory of the computer, using a software developed for this specific purpose *(Swistrack, Thomas Lochmatter, Switzerland)*. Cameras were also connected to battery packs and dedicated synchronization boxes (Meyer et al., 2012). These boxes use *GNSS* signal to achieve wireless synchronization of the cameras recording system and ensure images from the six cameras are taken simultaneously with an error of less than 2.00 μs.

The athletes used their own *GS* skis to completed three trials of the *GS*. The runs were recorded and the time needed to go through three considered gates was estimated by counting the number of images captured on video. The fastest run of each skier was then analyzed (typical speed around 20 m/s). The selected runs were processed with SIMI motion software *(SIMI motion, SIMI, Germany)*, using the panning and tilting modules. The camera’s internal (e.g., focal length, image format and principal point) and external (e.g., camera position and orientation) parameters needed for the analysis were determined using the DLT 11 calibration method (Hatze, 1988; Abdel-Aziz and Karara, 2015).

Participants had to wear a white racing suit previously equipped with 14 black markers, a black helmet, and black gloves. *CoM* of both ski poles were also marked with black markers. In total, 19 markers were identified, and 3D models composed of 14 segments were built (Figure 1B). The *CoM* of the skier was calculated using the model proposed by Clauser et al. (1969) modified to take the material’s weight into account. Tree morphological points that could be used for further analysis (e.g., *GNSS* antenna placement) were also defined: The *Pelvis* position was defined as the middle point between the 2 trochanters’ markers, the *Feet* position as the middle point between the 2 ankle-bone markers and the *Head* position as the center of the helmet.

The accuracy of the reconstruction method was measured in two different ways. First, the positions of three gates as given by the total station were compared with the positions calculated by the software. Second, the error in the length of each body segment was determined.

### Parameters Analysis

The Δ*Epot* (J/kg), *Ekin* (J/kg), and *Trad* (1/m), were calculated for the *CoM* and the three morphological points *(i, with i = {CoM, Head, Pelvis, Feet})*. For analysis purposes, each trial was normalized to fit a 100% temporal turn cycle, where 0 and 100% were the time points when the projection of the *CoM* was between the two skis. A cubic B-splines interpolation method was used to achieve the normalization (Greville, 1964; Lee et al., 1997).

#### Potential Energy

As the different morphological points are positioned at different heights of the body, the Δ*Epot*_i_ was calculated at each percent of the turn cycle, using the mass of the skier including equipment *(M)*, the acceleration due to gravity *(g)* and the delta height *(*Δ*H*_i_*(t))* of the analyzed point in a global reference system:

(1) $$\Delta {\mathrm{Epot}}_{\text{i}}(t)=M\cdot g\cdot \Delta H_{\text{i}}(t)$$

#### Kinetic Energy

The *Ekin*_i_ evolution during the turn was calculated using the speed *(V*_i_*)* of the analyzed points (calculated as the time derivative of the point coordinate) and *M*, using the following equation:

(2) $${\mathrm{Ekin}}_{\text{i}}(t)=0.5\cdot M\cdot V_{\text{i}}{\left(t\right)}^{2}$$

The mean *Ekin (Ekin*_m_*)* was also calculated over the whole turn cycle, to show the overall error when using a morphological point instead of the *CoM*.

#### Turn Radius

The *Trad*_i_ were calculated directly with SIMI motion, using the Frenet-Serret formula (Serret, 1851; Frenet, 1852). The turn entry *(Tentry*_i_*)* was defined as the instant where the *Trad*_i_ dropped below the natural radius of the skis (i.e., 25 m) and the turn exit *(Texit*_i_*)* as the instant where the turn radius went over 25 m again.

### Statistical Analysis

Statistical parametric mapping (*SPM*) was used on paired Student *T*-Tests (Pataky, 2010; Pataky et al., 2015) to analyze data over the whole turn cycle, to compare Δ*Epot*, *Ekin* and *Trad* obtained for the morphological points to the *CoM* reference. The fit between the curves was assessed by summing percent of time of a turn cycle where *SPM* indicate significant differences (*tt* values with *p* < .05). Paired Student *T*-tests were also used to assess statistical differences between the *CoM* and the morphological points for the *Ekin*_m_, *Tentry*, and *Texit*, given as mean ±*SD*. For all statistical analyses, significance was accepted at *p* < .05.

## Results

### 3D Accuracy

For the global gates position reconstruction using the 3D reconstruction software, a horizontal mean error of 14.0 ± 8.0 mm was calculated, giving a 95% limit of agreement of 27.1 mm. For the vertical error, the absolute mean of 5.9 ± 3.5 mm gave a 95% limit of agreement of 11.6 mm. Adding the horizontal and the vertical errors led to a total 3D reconstruction error of 15.7 ± 7.8 mm, and a 95% limit of agreement of 28.3 mm. The segments lengths mean error of 13.0 ± 12.0 mm led to a 95% limit of agreement of 32.7 mm.

### Potential Energy

**FIGURE 2:**
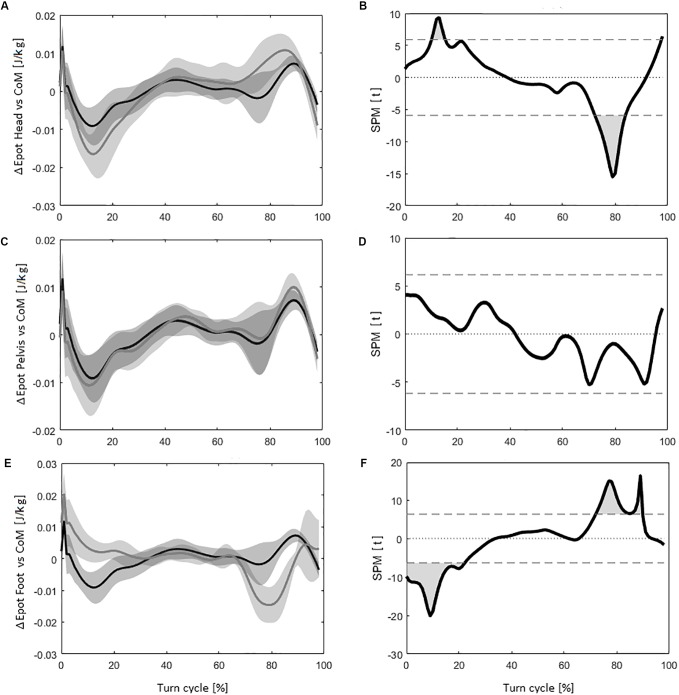
**(A)** The evolution of the delta of potential energy (Δ*Epot*) for the *CoM* (in black) and the *Head* (in dark gray) during the turn cycle, with the corresponding standard deviation (*SD*) (in light gray). **(B)** Evolution of statistical parametric mapping (*SPM)* values (*tt*) during the turn cycle, with light gray area representing the portions of the turns were it is statistically possible to differentiate the *CoM* with the *Head* (the horizontal dash lines correspond to *tt* = 6.79, *p* = 0.05). **(C)** The evolution of Δ*Epot* for the *CoM* (in black) and the *Pelvis* (in dark gray) during the turn cycle, with the corresponding *SD* (in light gray). **(D)** Evolution of *SPM* values (*tt*), with light gray area representing the portions of the turns were it is statistically possible to differentiate the *CoM* with the *Pelvis.* **(E)** The evolution of Δ*Epot* for the *CoM* (in black) and the *Feet* (in dark gray) during the turn cycle, with the corresponding *SD* (in light gray). **(F)** Evolution of *SPM* values (*tt*), with light gray area representing the portions of the turns were it is statistically possible to differentiate the *CoM* with the *Feet*.

Using the *Head* instead of the *CoM* to estimate Δ*Epot* led to significantly different values for 12% of the time course of the turn during the turn cycle. The corresponding curves are plotted on Figure 2A with the corresponding *SPM* results on Figure 2B (*tt* threshold at ±5.90).

When the *Pelvis* was used instead of the *CoM* to calculate Δ*Epot*, no significantly different values were obtained. Figure 2C shows the evolution of the Δ*Epot* between the *CoM* and the *Pelvis* with the corresponding *SPM* curve on Figure 2D (*tt* threshold at ±6.16).

Concerning the use of the *Feet* to estimate the Δ*Epot*, 41% of the measures during the turn cycle had significantly different values (*tt* threshold at ±6.33) compared to the values obtained using the *CoM*, as seen in Figures 2E,F.

### Kinetic Energy

**FIGURE 3:**
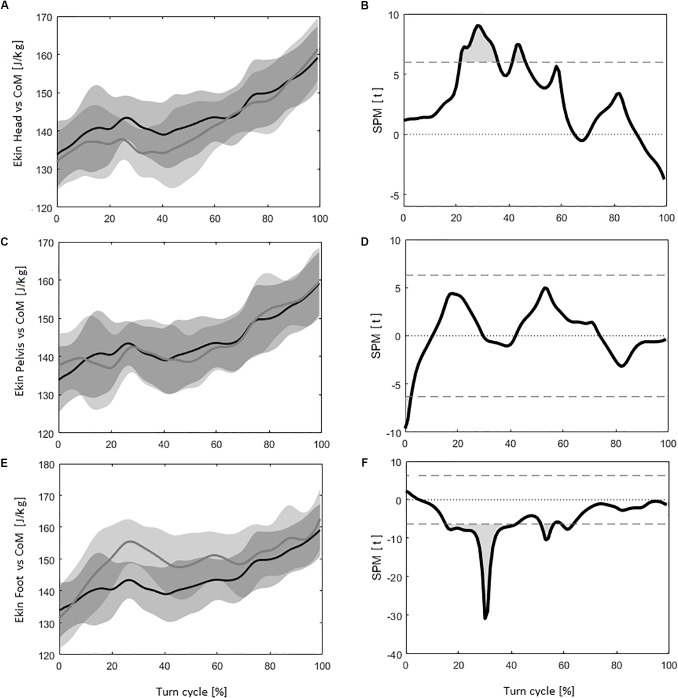
**(A)** The evolution of the kinetic energy (*Ekin*) for the *CoM* (in black) and the *Head* (in dark gray) during the turn cycle, with the corresponding standard deviation (*SD*) (in light gray). **(B)** Evolution of statistical parametric mapping (*SPM*) values (*tt*) during the turn cycle, with light gray area representing the portions of the turns were it is statistically possible to differentiate the *CoM* with the *Head* (the horizontal dash lines correspond to *tt* = 6.79, *p* = 0.05). **(C)** The evolution of *Ekin* for the *CoM* (in black) and the *Pelvis* (in dark gray) during the turn cycle, with the corresponding *SD* (in light gray). **(D)** Evolution of *SPM* values (*tt*), with light gray area representing the portions of the turns were it is statistically possible to differentiate the *CoM* with the *Pelvis.* **(E)** The evolution of *Ekin* for the *CoM* (in black) and the *Feet* (in dark gray) during the turn cycle, with the corresponding *SD* (in light gray). **(F)** Evolution of *SPM* values (*tt*), with light gray area representing the portions of the turns were it is statistically possible to differentiate the *CoM* with the *Feet*.

From the *Ekin* calculation, it can be seen that the *Head* induced significantly different values for 20% of the measures during the turn cycle compared to the results obtained using the *CoM*. Figure 3A represents the evolution of the curves during the turn cycle, while Figure 3B shows the corresponding SPM results (*tt* threshold at ±5.99). Compared to the *CoM*, the *Head* induced a significant underestimation of −2.57 ± 1.22 J/kg (*p* < 0.001) when calculating *Ekin*_m_.

Compared to the *CoM*, The *Pelvis* induced significantly different values for only 2% of the *Ekin* measurement during the turn cycle (Figures 3C,D) (*tt* threshold at ±6.31). No significant difference were found between the *CoM* and the *Pelvis* for *Ekin*_m_ (−0.22 ± 0.93 J/kg, *p* = 1.000).

For the *Feet*, *Ekin* calculation led to significantly different values for 36% of the turn cycle compared to the *CoM*. Figures 3E,F displays the evolution of the *Ekin* curve and *SPM* results (*tt* threshold at ±6.29). The *Feet* induced a significant overestimation of *Ekin*_m_ [5.77 ± 4.00 J/kg (*p* < 0.001)] compared to the result obtained with the *CoM*.

### Turn Radius

**FIGURE 4:**
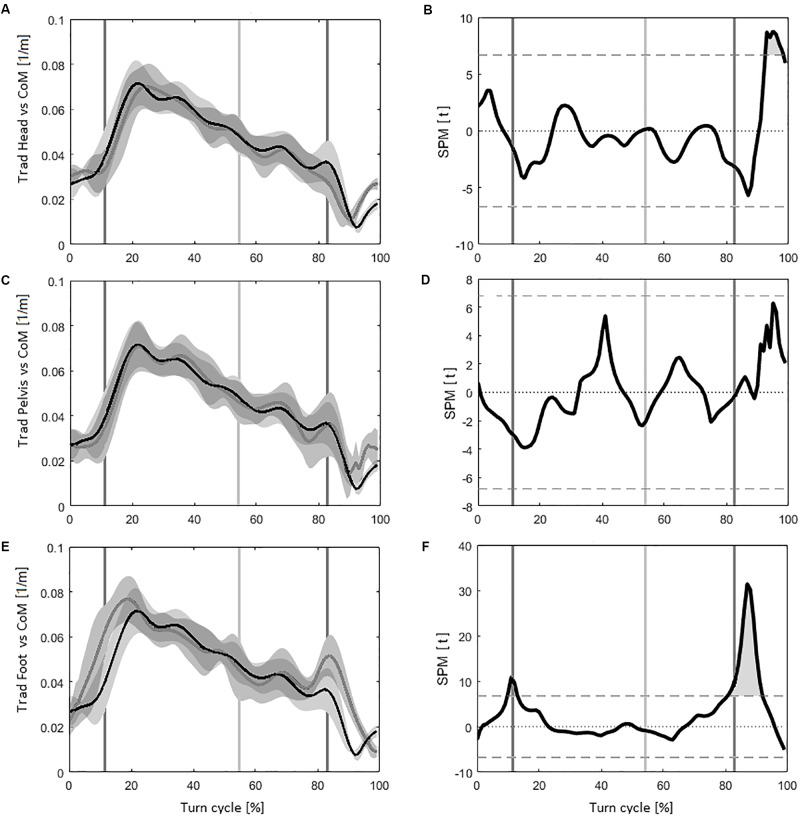
**(A)** The evolution of the turn radius (*Trad*) for the *CoM* (in black) and the *Head* (in dark gray) during the turn cycle, with the corresponding standard deviation (*SD*) (in light gray). **(B)** Evolution of statistical parametric mapping (*SPM*) values (*tt*) during the turn cycle, with light gray area representing the portions of the turns were it is statistically possible to differentiate the *CoM* with the *Head* (the horizontal dash lines correspond to *tt* = 6.79, *p* = 0.05). **(C)** The evolution of *Trad* for the *CoM* (in black) and the *Pelvis* (in dark gray) during the turn cycle, with the corresponding *SD* (in light gray). **(D)** Evolution of *SPM* values (*tt*), with light gray area representing the portions of the turns were it is statistically possible to differentiate the *CoM* with the *Pelvis.* **(E)** The evolution of *Trad* for the *CoM* (in black) and the *Feet* (in dark gray) during the turn cycle, with the corresponding *SD* (in light gray). **(F)** Evolution of *SPM* values (*tt*), with light gray area representing the portions of the turns were it is statistically possible to differentiate the *CoM* with the *Feet*.

The results obtained using the *Head* instead of the *CoM* for the calculation of *Trad* indicated significant differences for 7% of the turn cycle. Evolution of *Trad* is described in Figure 4A, with the corresponding SPM values Figure 4B (*tt* threshold at ±6.67).

The calculation of *Trad* using the *Pelvis* instead of the *CoM* induced no significant differences during the whole turn cycle (*tt* threshold at ±6.79). The corresponding curves are plotted on Figures 4C,D.

Using the *Feet* instead of the *CoM* to estimate *Trad* revealed that 16% of the measures had significantly different values during the turn cycle. Figures 4E,F displays the evolution of the *Trad* curve and *SPM* results for the comparison of the *CoM* and the *Feet* during the turn cycle (*tt* threshold at ±6.76).

Comparison of *Tentry* and *Texit* between the *CoM* and the morphological points can be found in Table 1.

**Table 1:** Moment of the turn cycle (in %) when the radius falls below 25 m (Tentry) and exceed 25 m again (Texit).

	Tentry mean ± SD [%]	Texit mean ± SD [%]
CoM	12.33 ± 2.88	84.67 ± 2.58
Head	14.50 ± 3.02	74.67 ± 4.64^∗^
Pelvis	13.17 ± 3.19	13.17 ± 3.19
Feet	6.50 ± 3.02^†^	90.50 ± 1.98^†^

Paired T-Test comparing CoM to Head, Pelvis and Feet. ^∗^p < 0.001, ^†^p < 0.05.

## Discussion

The most important finding of this study was the high level of agreement between the *Pelvis* and the *CoM*. Indeed, when looking at the different parameters analyzed, the *Pelvis* offered the best estimation for the Δ*Epot*, *Ekin* and *Trad* calculation. No significant differences were found for the Δ*Epot* and *Trad* during the whole turn whilst only 2% of the turn cycle significantly differed in the case of the *Ekin*. The difference was encountered only at the beginning of the turn.

As a global observation, it is quite intuitive to see the *Feet* and the *Head* as extreme points of the skier, while the *Pelvis* is more centered and near the *CoM*. Nevertheless, the *Head* allowed slightly better estimations than the *Feet* for the analyzed parameters showing more similar patterns of the *CoM*. The angulation of the hips during the second half of the turn can probably explain this result, as the *Head* is more centered vertically on the *CoM* while the *Feet* follow an external trajectory. The best morphological point to estimate Δ*Epot* and *Ekin* is therefore the *Pelvis*, followed by the *Head* and finally by the *Feet* that offer poor reliability.

### Energy

As *Epot* is directly correlated to vertical displacement, the curves of Δ*Epot* obtained in this study can be compared to the work proposed by Pozzo et al. (2005), who calculated the vertical displacement of the *CoM* compared to the ground. As expected, the *CoM* was higher during transitions between turns and lower at gate crossings. This corresponds well to the interpretation of Δ*Epot* curves calculated using the *CoM* and the *Feet* in the present study.

As the *Ekin* values depend on the square power of the speed, the shape of the curves obtained in this study can also be compared to those obtained by Pozzo et al. (2005) for the speed of the skiers during the turns. The measured speed attained its maximal value during gate transition, as it does in the present study.

**FIGURE 5:**
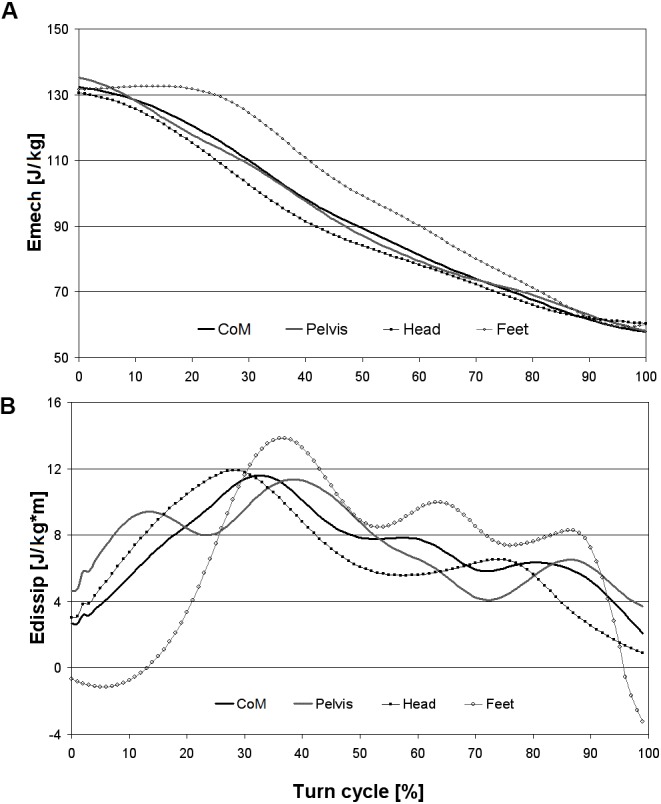
**(A)** Mechanical energy (*Emech*) calculated using the CoM and the morphological points, **(B)** Energy dissipation (*Edissip*) during the turn.

Supej (2008) and Reid et al. (2009) analyzed the mechanical energy of skiers *(Emech)*, which involved addition of the *Ekin* and the *Epot*. They also calculated the corresponding dissipated energy *(Edissip)* as the change in mechanical energy per change of vertical distance (Supej et al., 2005). To allow comparison with these studies, Figures 5A,B show the *Emech* and the *Edissip* respectively, calculated using the *CoM* and the morphological points of the present study.

The curves obtained for the *CoM* are very similar to those obtained by Supej (2008) in *GS* and Reid et al. (2009) in slalom. The minimum energy dissipation occurred at the turn transition and the maximum during the first steering phase, between 20 and 40% of the turn cycle.

### Turn Radius

The *Trad* described by the *Feet* trajectory began earlier and ended after the *Trad* of the *CoM*. The *Head* also finished the turn earlier than the *CoM*. Therefore, the Head had the longer time interval between two turns where its trajectory was almost straight, and the Feet had the shortest time interval with a straight trajectory. It was interesting to note that around the gate crossing, inter-athlete variability increased, suggesting that the gates induced perturbation. If the radius decreased during the transition phase to reach its minimum, it increased gradually during the steering phases. Supej (2008) obtained a curve of a similar shape when calculating the *CoM*’s turn radius of four athletes performing *GS*. For slalom turns, Reid et al. (2009) obtained a different curve in slalom, where the radius decreased slowly during the first part of the turn and increased rapidly at the end of the turn. This indicates a different choice of trajectory in giant compared to slalom.

The *Feet* trajectory radii showed a small reduction between the second steering phase and the transition phase, when the skier decided to engender the new turn. It was at this same moment that the skier made a longitudinal extension, when the *Epot_diff* between the *CoM* and the *Head* increased, at approximately 80% of the turn cycle (Figure 3A).

Once again, the *Pelvis* gave the best approximation of the *CoM* concerning turn radius, followed by the *Head*. The *Feet*, with a time lag in the turn radius did not offer a good approximation of the *CoM*’s *Trad*, but it could be interesting to further explore the radius reduction around 85% of the turn. Indeed, it may be possible that this radius reduction coincides with an increase in the force and an extension of the skier to trigger the next turn.

## Conclusion

It is the first time that different morphological points of the body are used to estimate energetic parameters of alpine skiers. The results obtained with the *Pelvis* offered very accurate approximations of the *CoM*, with an equivalent accuracy than the pendulum method used by Gilgien et al. (2013). The *Head* also offered a good approximation for overall energy analysis and is a very accessible point for 3D video tracking or *GNSS* antenna placement, but side leaning profiles induced inaccurate estimations in the middle part of the turn. Finally, the *Feet* did not allow for a good estimation of the *CoM* as most of the parameters did not even have curves that look like those described by the *CoM*.

## Data Availability Statement

The raw data supporting the conclusions of this manuscript will be made available by the authors, without undue reservation, to any qualified researcher.

## Ethics Statement

This study was carried out in accordance with the recommendations of the HRO Guidelines from the “Commission cantonale (VD) d’éthique de la recherche sur l’être humain.” The protocol was approved by the “Commission cantonale (VD) d’éthique de la recherche sur l’être humain.” All subjects gave written informed consent in accordance with the Declaration of Helsinki.

## Author Contributions

All authors listed have made a substantial, direct and intellectual contribution to the work, and approved it for publication.

## Conflict of Interest Statement

The authors declare that the research was conducted in the absence of any commercial or financial relationships that could be construed as a potential conflict of interest.
